# The Prevalence of Bilateral Three-Rooted Mandibular First Molar in Indian Population

**Published:** 2013-08-01

**Authors:** Rupali Karale, Champa Chikkamallaiah, Jayshree Hegde, Srirekha Aswathanarayana, Lekha Santhosh, Kusum Bashetty, Shwetha Rajanna Susheela, Srinivas Panchajanya

**Affiliations:** a Department of Conservative Dentistry and Endodontics, Oxford Dental College, Bommahalli, Bangalore, India

**Keywords:** Endodontic, Mandibular, Molars, Root canal, Three-Rooted

## Abstract

**Introduction:**

The purpose of the present study was to evaluate the prevalence of bilateral three-rooted mandibular first and second molars in Indian population.

**Materials and Methods:**

A total of 215 patients were screened bilaterally for mandibular first and second molar and 430 samples of periapical radiographs were obtained. The gender, symmetry, and prevalence of three-rooted mandibular first and second molars were recorded. The correlation between left and right side occurrences and distribution were recorded and analysed using Z-test.

**Results:**

The results showed that 33 teeth had three-rooted mandibular first molars, 16 male and 17 female (P=0.442). Overall, 21 teeth of right jaw and 12 teeth of left jaw (P=0.103) showed presence of an extra-root. The prevalence of three-rooted mandibular first molars was 7.67% and second molar was 0.23%. The bilateral frequency distribution was 3.72% for the first molar. There was no statistically significant difference between right side and left side mandibular molars. Also, gender did not show a significant relationship with this variant.

**Conclusion:**

The endodontic treatments of first mandibular molars require a careful clinical approach in Indian population as a high racial prevalence of 7.67% three-rooted molars was found. However, in the same population, 0.23% mandibular second molars had three roots.

## Introduction

The prevention or healing of endodontic pathology depends on a thorough chemo-mechanical cleansing and shaping of the root canals before a dense root canal filling with a bacteria-tight seal is placed. An awareness and understanding of the presence of unusual root canal morphology can thus contribute to the successful outcome of root canal treatment. The mandibular first molar can display several anatomical variations. The major variant is the occurrence of a third root, which is well documented in the literature. This supernumerary root located distolingually, first mentioned by Carabelli [1], called radix entomolaris [2]. An additional root at the mesiobuccal side is called radix paramolaris (RP) [2-4].

This additional root shows a frequency of less than 4% in whites and about 2.8% in African populations, whereas in population with mongoloid traits (American Indians, Eskimos, and Chinese) the frequency is between 5% and 30% [4, 5].

In these populations, the radix entomolaris is seen as a normal morphologic variant [3] and can be seen as an Asiatic trait [6]. According to Quackenbush the extra root occurred unilaterally in approximately 40% of all cases and predominately on the right side [7]. Although these observations showed that radix entomolaris could be related to penetrance of an atavistic gene or polygenetic system, the etiology is unclear. Besides racial genetic factors, external factors during odontogenesis might also be responsible for additional roots and has reflected a high degree of genetic penetrance as its dominance in the pure Eskimo and Eskimo/ Caucasians mixes had similar prevalence of the trait [8, 9]. 

Among the research activities done on prevalence of radix entomolaris on mandibular molar teeth very few studies have been reported on bilateral occurrence of RE in both first and second mandibular molars. Therefore the purpose of this study was to evaluate the prevalence of bilateral radix entomolaris/paramolaris and gender difference of three-rooted mandibular molars in Indian population.

## Material and Methods

Total of 215 patients of known racial origin from all age groups who presented to the Department of Conservative Dentistry and Endodontics, were screened for bilateral intra-oral periapical radiographs. Patients with missing unilateral molars were excluded from the study. The study protocol was approved by the Ethics Committee of the Oxford Dental College (No.315/2011-12). Patients with unilateral intra-oral periapical (IOPA) radiograph were advised to take contralateral PA’s to achieve bilateral IOPA with required informed consent. Periapical radiographs were taken by means of a Rinn XCP (Dentsply, Elgin, IL, USA) for each patient. The x-ray machine used was a Heliodent DS (Sirona Factors, Bensheim, Germany) (7mA and 60kV). Periapical films (Eastman Kodak ultra-speed film; Kodak Rochester, NY, USA) were used for image retention. 

Periapical radiographs of a total of 215 patients showing bilateral mandibular first molars and second molars were chosen for this investigation. Radiographs were inspected by two endodontists/authors (RK and CC) using a magnifying view box. The criteria for the indication of an extra-root were justified by the crossing of the translucent lines defining the pulp space and periodontal ligament in mandibular first and second molar teeth. Disagreement in the interpretation of radiographs was discussed between two investigators until a consensus was reached. The total prevalence of three-rooted mandibular first and second molars and the ratio of the occurrence in gender of such teeth were assessed. The prevalence of bilateral and unilateral three-rooted molars and the comparison of right to left side were analysed by using Z-test.

## Results

A total of 215 patients, 118 males and 97 females, aged between 16 and 60 years were considered in this study. A total of 430 mandibular molars were evaluated.

Out of 430 mandibular first molars, 33 teeth showed presence of an extra-root. The overall prevalence of teeth showing an extra-root from all teeth examined was 7.67%. Eight patients showed bilateral presence of extra-roots with a prevalence of 3.72%. Only one mandibular second molar showed presence of three roots (0.23%) (Table 1). Twenty one right molars and twelve left molars showed presence of an extra-root (*p*=0.103). Sixteen out of 236 teeth of male patients and seventeen out of 194 teeth of female patients examined showed an extra-root (*p*=0.442) (Table 2).

**Table 1.** Analysis of distribution of three roots in the samples examined

Mandibular first molar featuring three roots	N	%
**Bilateral**	8	3.72
**Total teeth featuring extra-root**	33	7.67
**Total teeth examined**	430	

**Table 2.** Test of significance for frequency of distribution: [Z-test for proportions, P> 0.05]

	Examined Teeth (n)	Three-rootedTeeth (n)	Z-test	*P*-value
**Male**	236	16	-0.77	0.442
**Female **	194	17		
**Right **	215	21	1.63	0.103
**Left **	215	12		

## Discussion

The dental surgeon must be aware of racial anatomical variations since he may see patients of diverse origins in his or her practice. The present study investigated prevalence of three-rooted mandibular molars in an Indian population using a periapical radiographic method and found a significant percentage of 7.67% patients with an extra third root in mandibular first molar and 0.23% in mandibular second molar.

The prevalence in our study was higher than the study carried out by Tratman in Indian population, in 1938 who examined 453 teeth with 0.2% incidence of three-rooted mandibular first molar [10]. However a more recent study by Garg *et al*. who examined 1054 periapical radiographs, reported 5.97% of occurrence of RE in mandibular first molars [11]. These contradictory findings may be explained by marked differences in the sample size/methods used.

The frequency of such dental aberrations according to gender did not appear to show a statistically significant difference in our study. The prevalence of the extra disto-lingual root for first mandibular molar on right side was more than left side but this was not statistically significant. We found that the bilateral prevalence of three rooted mandibular molars was 3.72%. This result was les than the reported figures of 56.6-67% of several researches on Far East Asians (those of Japanese and Chinese descent) [6, 12]. Schafer *et al*. has reported only unilateral occurrence of supernumerary root [13]. This inter study variation may relate to sample size, racial difference and case selection. Further investigations may be necessary to clarify the issue.

An additional root may contribute to localized periodontal destruction and create greater probing depths and attachment loss at the distolingual sites in addition to the well-known endodontic problems associated with a “missed” canal [14]. In case of supernumerary root, the conventional triangular access cavity must be modified to a trapezoidal form with extension to the disto-lingual. When the disto-lingually located additional canal orifices have been located, the creation of a straight line access is of utmost importance because most of the additional roots are severely curved [3, 8].

The actual incidence of radix entomolaris is based on extracted teeth examined for racial difference. It is therefore impossible to make inter study comparisons relating to gender and bilateral occurrence differences for such three-rooted mandibular first molars from extracted teeth, unless detailed recordings were done before extraction [15, 16].

However, availability of extracted teeth for research purposes has progressively declined due to improvements in general dental health. Clinically, use of radiographs can be a useful aid for research purpose [3, 8, 17].

The use of periapical radiographic method is non-invasive and allows for inter-study comparisons relating to gender and bilateral occurrence difference for three-rooted mandibular first molar [9, 18, 19]. The two- dimensional radiograph image from this investigation technique and the film-processing quality might both constitute concerns as regards to the study of 3-D tooth roots. Clinical oral examination with periodontal probing and a review of patient personal dental records combined with either two/three dimensional radiographic technique like microcomputed tomography may be more accurate method for investigating prevalcence tooth anomaly in different ethnic groups.[20]. 

A radix entomolaris can be found on the first, second and third mandibular molars occurring least frequently on the second molars [19]. Some studies reported occurrence of RE from 50 to 67% [6, 12]. Bolk (1915) reported the occurrence of a buccally located additional root, the RP. This macro-structure is very rare and occurs less frequently than the [2]. The prevalence of RP, as observed by Visser was found to be 0% for the first mandibular molar, 0.5% for the second and 2% for the third molar [21]. Other studies have, however reported RP in first mandibular molar [4, 22]. Our study demonstrated the prevalence of 0.23% of RE in mandibular second molar.

The previous knowledge and diagnosis of supernumerary roots can avoid complication such as missed canals during endodontic treatment. Diagnosis can be made by thorough radiographic examination, clinical inspection of crown-extra-cusp (tuberculum paramolare), analysis of cervical morphology of roots by means of periodontal probing, use of visual aids such as a loop, intra-oral camera, dental microscope, etc. 

A study on root canal morphology of RE using micro-CT scanning has reported that vertical length, width, bucco-lingual/mesio-distal diameter ratio, wall thickness and bucco-lingual taper of the disto-buccal canals are generally greater than the disto-lingual canals. The mesio-buccal, mesio-lingual and disto-buccal canals are more oval, whereas the disto-lingual canals are relatively rounder. As unique anatomic features affects root canal preparation and removal of the debris, the clinicians must be aware of the unusual anatomic morphology of the roots [23].

While treating RE the knowledge of mean inter-orifice distances might help dentists to locate orifices and to achieve successful endodontic treatment. Tu *et al*. has reported that mean inter-orifice distances from the disto-lingual canal to the disto-buccal, mesio-buccal and mesio-lingual canals of the permanent three-rooted mandibular molars were 2.7, 4.4 and 3.5 mm, respectively [24]. Apart from knowing the prevalence of extra-root, further studies are required to know the detailed root canal morphology of extra root to locate and prepare canals for long term function.

## Conclusion

Based on the obtained results, the relatively high racial prevalence of third-root in mandibular first molars in Indian patients makes thorough knowledge of unusual root morphologies mandatory for clinicians.

## References

[1] Carabelli, G (1844). Systematisches Handbuch der Zahnheilkunde,.

[2] Bolk L (1915). Bemerkungen über Wurzelvariationen am menschlichen unteren Molaren.. Zeitschrift für Morphologie und Anthropologie..

[3] Calberson FL, De Moor RJ, Deroose CA (2007). The radix entomolaris and paramolaris: clinical approach in endodontics.. J Endod..

[4] Sperber GH, Moreau JL (1998). Study of the number of roots and canals in Senegalese first permanent mandibular molars.. Int Endod J..

[5] Ferraz JA, Pecora JD (1993). Three-rooted mandibular molars in patients of Mongolian, Caucasian and Negro origin.. Braz Dent J..

[6] Yew SC, Chan K (1993). A retrospective study of endodontically treated mandibular first molars in a Chinese population.. J Endod..

[7] Quackenbush LE (1986). Mandibular molar with three distal root canals.. Endod Dent Traumatol..

[8] De Moor RJ, Deroose CA, Calberson FL (2004). The radix entomolaris in mandibular first molars: an endodontic challenge.. Int Endod J..

[9] Curzon ME (1974). Miscegenation and the prevalence of three-rooted mandibular first molars in the Baffin Eskimo.. Community Dent Oral Epidemiol..

[10] Tratman E (1938). Three-rooted lower molars in man and their racial distribution.. Br Dent J..

[11] Garg AK, Tewari RK, Kumar A, Hashmi SH, Agrawal N, Mishra SK (2010). Prevalence of three-rooted mandibular permanent first molars among the Indian Population.. J Endod..

[12] Steelman R (1986). Incidence of an accessory distal root on mandibular first permanent molars in Hispanic children.. ASDC J Dent Child..

[13] Schafer E, Breuer D, Janzen S (2009). The prevalence of three-rooted mandibular permanent first molars in a German population.. J Endod..

[14] Huang RY, Lin CD, Lee MS, Yeh CL, Shen EC, Chiang CY, Chiu HC, Fu E (2007). Mandibular disto-lingual root: a consideration in periodontal therapy.. J Periodontol..

[15] Jones AW (1980). The incidence of the three-rooted lower first permanent molar in Malay people.. Singapore Dent J..

[16] Curzon ME (1973). Three-rooted mandibular permanent molars in English Caucasians.. J Dent Res..

[17] Goerig AC, Neaverth EJ (1987). A simplified look at the buccal object rule in endodontics.. J Endod..

[18] Tu MG, Tsai CC, Jou MJ, Chen WL, Chang YF, Chen SY, Cheng HW (2007). Prevalence of three-rooted mandibular first molars among Taiwanese individuals.. J Endod..

[19] Walker RT (1988). Root form and canal anatomy of mandibular first molars in a southern Chinese population.. Endod Dent Traumatol..

[20] Jin GC, Lee SJ, Roh BD (2006). Anatomical study of C-shaped canals in mandibular second molars by analysis of computed tomography.. J Endod..

[21] Visser JB (1948). Beitrag zur Kenntnis der menschlichen Zahnwurzelformen: Buchdruckerei Rotting.

[22] Carlsen O, Alexandersen V (1991). Radix paramolaris in permanent mandibular molars: identification and morphology.. Scand J Dent Res..

[23] Gu Y, Zhou P, Ding Y, Wang P, Ni L (2011). Root canal morphology of permanent three-rooted mandibular first molars: Part III--An odontometric analysis.. J Endod..

[24] Tu MG, Huang HL, Hsue SS, Hsu JT, Chen SY, Jou MJ, Tsai CC (2009). Detection of permanent three-rooted mandibular first molars by cone-beam computed tomography imaging in Taiwanese individuals.. J Endod..

